# Repurposing of F-gases: challenges and opportunities in fluorine chemistry

**DOI:** 10.1039/d1cs01072g

**Published:** 2022-05-26

**Authors:** Daniel J. Sheldon, Mark R. Crimmin

**Affiliations:** Department of Chemistry, Molecular Sciences Research Hub, Imperial College London 82 Wood Lane, Shepherds Bush London W12 0BZ UK m.crimmin@imperial.ac.uk

## Abstract

Fluorinated gases (F-gases) are routinely employed as refrigerants, blowing agents, and electrical insulators. These volatile compounds are potent greenhouse gases and consequently their release to the environment creates a significant contribution to global warming. This review article seeks to summarise: (i) the current applications of F-gases, (ii) the environmental issues caused by F-gases, (iii) current methods of destruction of F-gases and (iv) recent work in the field towards the chemical repurposing of F-gases. There is a great opportunity to tackle the environmental and sustainability issues created by F-gases by developing reactions that repurpose these molecules.

## Abstract 2

The applications, environmental issues, and methods for remediation of F-gases are reviewed.

## Graphical abstract

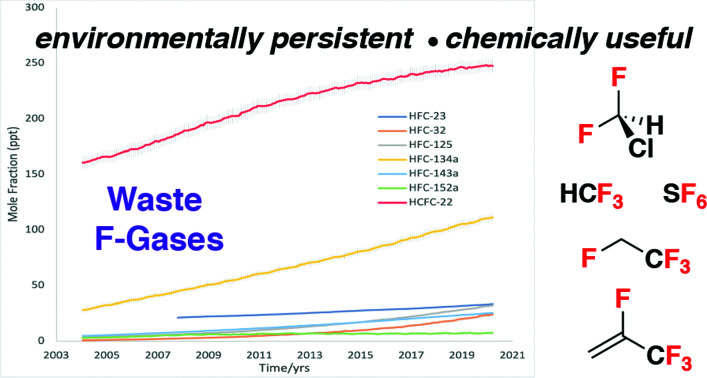

## 1. Introduction

**Fig. 1:**
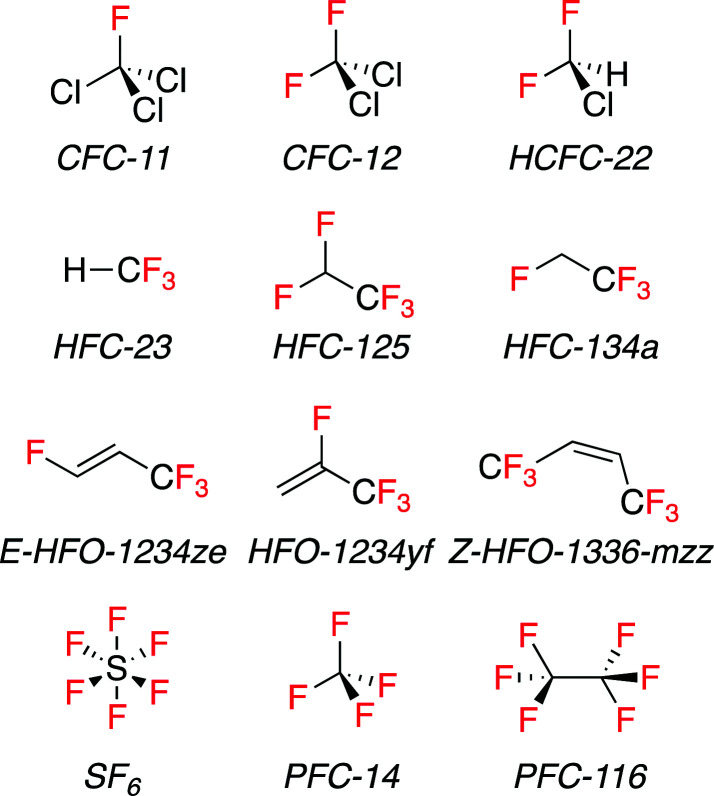
Line drawings of common F-gases.

Fluorinated compounds have greatly improved the quality of our life over the last century. They have found wide ranging uses in the refrigeration, pharmaceutical and agrochemical industries, and as propellants, surfactants, polymers, and fire suppressants. Due to its lack of polarizability fluorine does not engage in many intermolecular interactions, and so fluorinated compounds often have low surface energies and consequently low boiling points compared to their non-fluorinated analogues. This is true of F-gases. F-gases include chlorofluorocarbons (CFCs), hydrochlorofluorocarbons (HCFCs), hydrofluorocarbons (HFCs), perfluorocarbons (PFCs), and sulfur hexafluoride (SF_6_). They are a class of volatile molecules that are defined by the inclusion of at least one fluorine atom and their low boiling point, arguably hydrofluoroolefins (HFOs) can also be included in this compound class (Fig. 1).

Despite their widespread usage, there are almost no naturally occurring fluorocarbons. The majority of fluorinated chemicals, including F-gases, are made from inorganic fluoride (*i.e.* CaF_2_). Reaction of CaF_2_ with H_2_SO_4_ produces HF; a widely used chemical intermediate.^1^ HF can be converted into fluorocarbons predominantly through the Swarts process and the Balz–Schiemann process.^2,3^ HF can also be used to make elemental fluorine, F_2_, which can then be used to synthesise perfluorocarbons in the Fowler process.^4,5^ Other major uses of F_2_ include the manufacture of UF_6_ from UF_4_ and in the production of SF_6_.^6^ In 2007, it was estimated there is a 100 year supply of CaF_2_ if the current rate of usage continues.^5^ While it is likely that new sources – or alternatives such as cryolite (Na_3_AlF_6_) or fluorapatite (Ca_5_(PO_4_)_3_F) – will be found,^6^ the rate of consumption is predicted only to increase as living standards increase.^7^

Fluorocarbons are often treated as ‘single-use’. One of the largest mechanisms of loss to the environment is through F-gas emissions. This has created a major environmental problem as many F-gases are potent greenhouse gases. Fluorine containing molecules are also discarded as a mixture of solid waste from metal manufacturing and fertiliser.^6^ Negligible amounts of fluorine used in industry is recycled or reclaimed, creating a major issue of sustainability. We foresee a huge demand for repurposing the fluorine in current waste streams, particularly from emitted F-gases. In this review article, we set out the environmental impact of F-gases and discuss recent work in the field for the chemical repurposing of these compounds. Recent reviews can be found on the history of fluorocarbon refrigerants and their syntheses,^8–11^ and one containing a discussion of the developments towards reducing F-Gas emissions.^12^ A further review details the advances made in using fluorinated gases in continuous flow processes.^13^

## 2. Applications of F-gases

### 2.1 CFCs and HCFCs

The first wide industrial application of F-gases (Table 1) was realised with CFCs in the early 1930s.^14^ These gases were used as refrigerants due to their low toxicity, low flammability, high volatility and high chemical and thermal stability.^1,15^ CFCs also later became the main components of aerosol propellants and blowing agents. Chlorotrifluoromethane (CFC-11) and dichlorodifluoromethane (CFC-12) were the primary refrigerants alongside hydrochlorofluorocarbons (HCFCs) until the 1980s, with these gases being produced on a scale of one million tonnes per annum at the peak of their production.

CFCs have very long atmospheric lifetimes and diffuse into the stratosphere where they are decomposed by UV radiation.^6^ UV radiation homolytically cleaves the C–Cl bonds of CFCs, creating chlorine radicals. These react with ozone to create oxygen and chlorooxide radicals, which then regenerate chlorine radicals and therefore propagate the cycle.^1,16^ Ozone depletion causes a major environmental problem as the ozone layer protects the earth's surface from harmful UV radiation.^6^ The Montreal Protocol agreed a phase out of CFCs from developed countries by 1996 and from developing countries by 2010.^17^ It is considered one of the most successful responses to a global environmental issue. HCFCs were seen as a temporary replacement for CFCs as they have a shorter atmospheric lifetime and contribute less to ozone depletion. Chlorodifluoromethane (HCFC-22) has an ozone depleting potential (ODP) about 20 times less than CFC-12, and so it became a popular replacement.^17^ However, HCFCs are still ozone-depleting substances and are also being phased out. By 2030, all HCFC consumption should be halted in most developed countries, and by 2040 for developing countries.^18,19^ However, production of HCFCs (such as HCFC-22) as a feedstock chemical (*e.g.* for fluoropolymer synthesis) is permitted to continue, and as such HCFC-22 is still being produced in large amounts as a chemical intermediate by fluoropolymer and polyfluoroalkyl substance (PFAS) manufacturing plants.^20^ A recent report from the US environmental protection agency (EPA) suggests the release of this gas to the environment may still be a serious issue.^21^

### 2.2 HFCs

The Montreal Protocol accelerated the need to find direct replacements for CFCs and HCFCs. Hydrofluorocarbons (HFCs) were identified because they do not deplete ozone, while they have similar chemical and thermal properties to CFCs.^16,22^ Nowadays, the majority of HFC consumption is for refrigeration purposes, with the most common being 1,1,1,2-tetrafluoroethane (HFC-134a).^17,23^ While the use of HFCs was seen to ‘fix’ the issue of ozone depletion, they brought a new issue to light in that they are greenhouse gases, absorbing infrared radiation in the region 1000–1400 cm^−1^. Global warming potential (GWP) is a metric that considers the amount of energy 1 ton of an emitted gas will absorb over a given period relative to 1 ton of emitted CO_2_ (often considered over 100 years, denoted GWP_100_). Most HFCs have a GWP_100_ over 1000 and are therefore considered highly potent greenhouse gases. The Kyoto Protocol, signed in 1997, committed signatories to reduce greenhouse gas emissions with a focus on the reduction of F-gases including HFCs, PFCs and SF_6_.^24,25^ However, it faced criticism over its narrow time period and the fact it focused mainly on developed countries.^24^ In order to reduce greenhouse gas emissions, further stringent restrictions on the production and use of HFCs were set, although only many years later. In 2016, the Kigali Amendment to the Montreal Protocol was signed which committed signatories to ‘phase-down’ HFCs, *i.e.* reduce the production and consumption of HFCs.^26–29^ Other legislation included the EU F-gas regulations, which further sought to limit fluorinated gas emissions by banning fluorochemical refrigerants with a GWP_100_ exceeding 150 in automobile air conditioning systems, effecting a ban on HFC-134a in its largest and most emissive application.^19,29^ It also banned the marketing of refrigeration and air conditioning equipment using these refrigerants, and required periodic inspection of stationary systems using HFCs.^30,31^

Alternative uses for HFCs are in applications such as blowing agents for producing foam plastics applied to insulation and packaging, aerosols in metered dose inhalers, fire suppressants and in the dry etching of dielectric films and fabrication of thin-film devices.^22,32^

### 2.3 HFOs

The next generation of refrigerants set to replace HFCs include hydrofluoroolefins (HFOs). HFOs are considered promising alternatives due to their low GWP and suitable chemical and physical properties.^33^ For example, *E*-1,3,3,3-tetrafluoropropene (*E*-HFO-1234ze) and 2,3,3,3-tetrafluoropropene (HFO-1234yf) both have a GWP_100_ of less than 1.^19^ The inclusion of a C=C double bond within the F-gas leads to a more reactive compound, thus enabling a lower atmospheric lifetime and GWP_100_. The counterpoint is that this substitution also leads to lower stability and higher toxicity.^33^ It is likely that these chemicals will decompose close to the source of emission, which can lead to local pollution, along with concerns that the decomposition products may themselves possess a high GWP_100_. There are also some thermophysical related issues, such as the low evaporating pressure and small vaporisation enthalpy of *E*-HFO-1234ze resulting in a lower coefficient of performance and a lower volumetric refrigeration capacity than HFC-134a.^34^ In order for industry to create a refrigerant that possesses the ideal thermophysical properties and a low GWP_100_, refrigerant blends comprising various HFOs and HFCs have been marketed These are often designed to offer a compromise between flammability and GWP.^35^ For example R-449a (Opteon XP40 by Chemours) has a 1 : 1 : 1 : 1 composition by % wt of HFC-32 : HFC-125 : HFO-1234yf : HFC134a.^34,36,37^ Refrigerant blends can be designed to have particular properties, perhaps superior to other pure refrigerants, but each element of added complexity increases cost, the refrigerant charge, the potential for leaks, while also decreasing reliability.^14,19^

### 2.4 Perfluorinated F-gases

SF_6_ is widely used as an electrical insulating gas in circuit breakers, transmission lines, transformers, and substations. 80% of SF_6_ produced between 1996–2003 was consumed by electric utilities and equipment manufacturers for electric power systems.^38,39^ Some of its other uses include semiconductor processing, a blanket gas for magnesium refining, thermal and sound insulation.^40^ It is a synthetic gas whose unique chemical and physical inertness (non-flammable, non-toxic, non-explosive) and excellent thermal conductivity mean it is ubiquitous in electrical insulation and largely irreplaceable. It is one of the most potent greenhouse gases and as such its emission creates a significant contribution to climate change, as will be discussed in further detail below. It was included in the Kyoto Protocol and EU F-gas regulations for limiting the emissions of fluorinated greenhouse gases.^25,30^ There are no restrictions on using SF_6_ in switchgear under these regulations, but there are requirements to recover SF_6_ where possible.^30,38^ The EPA established an agreement in 1995 with electric power systems to reduce emissions and in 2016 it was reported that the industrial partners who signed up had reduced their emissions by 76%.^38,39^ Furthermore, very recently there has been research to provide alternative gases to SF_6_ such as perfluoroketones, perfluoronitriles and CF_3_I.^41–44^ Newer wind-turbine technology has resulted in some SF_6_-free equipment or even vacuum insulated switchgear.^38^

Perfluorocarbons find wide-ranging applications as specialist solvents, blood substitutes, textile finishers and fire suppressants.^45,46^ They are also used widely in the electronic industry, with applications ranging from semiconductor manufacturing, integrated circuit components and in the assembly of power electronics.^25^ They have low surface energies, high dielectric strength and are compressible. They are also potent greenhouse gases, with the fully saturated C_1_ to C_6_ perfluorocarbons included in the Kyoto protocol of 1997.^47^ Efforts to limit emissions are being carried out by several developed countries.^6^ In 1999, the World Semiconductor Council committed to the reduction of PFC emissions by 10% or greater by 2010 from the baseline levels of 1995.^48^ This included exploring alternative chemicals such as NF_3_, improving capture/recycle systems and the optimisation of existing processes and destruction methods.^49^

## 3. Production and environmental impact of F-gases

In general, atmospheric concentrations of F-gases are low (relative to CO_2_), parts per trillion, but their impact is significant. Since 1978, the Advanced Global Atmospheric Gases Experiment (AGAGE) have measured the atmospheric concentrations of synthetic greenhouse gases from remote stations.^50,51^ These remote stations observe air masses using gas chromatography-electron capture detection and gas chromatography-mass spectrometry techniques.^50,52,53^ Many of the data discussed below are reported from these measurements.^18,38,47^ Top-down global emission estimates are derived from atmospheric concentration measurements from AGAGE, using the two-dimensional AGAGE 12-box model.^18,54,55^ From 1990 onwards, developed countries have been required to report emissions of HFCs, PFCs and SF_6_ as part of the United Nations Framework Convention on Climate Change (UNFCCC). However, by these reports half of the world's HFC emissions were unaccounted for when compared to global emission estimates derived from AGAGE atmospheric measurements.^18,29,56^ The implication is that much of the world's F-gas emissions are unreported by UNFCCC. This is likely due, in part, to emissions from developing countries.^57,58^

**Table 1:** Common CFCs, HCFCs, HFCs, HFOs and PFCs. GWP_100_ values and atmospheric lifetimes are given as reported in the sixth assessment report adopted by the Intergovernmental Panel on Climate Change (2021).^59^ It is noted that CFCs and HCFCs are also ozone-depleting substances

F-Gas	Main uses	Atmospheric lifetime (years)	GWP_100_
CFC-11, CFCl_3_	Refrigerant	52	5560
CFC-12, CF_2_Cl_2_	Refrigerant	102	11 200
HCFC-22, CF_2_ClH	Refrigerant	11.9	1960
HFC-23, CF_3_H	By-product	228	14 600
HFC-32, CF_2_H_2_	Refrigerant blend component	5.4	771
HFC-125, C_2_F_5_H	Refrigerant blend component, fire suppressant	30	3740
HFC-134a, CFH_2_CF_3_	Refrigerant, foam blowing agent, fire suppressant and propellant	14	1530
HFC-143a, CF_3_CH_3_	Refrigerant blend component	51	5810
HFC-152a, CF_2_HCH_3_	Refrigerant blend component, foam blowing agent and aerosol propellant	1.6	164
HFO-1234yf, H_2_C=CFCF_3_	Refrigerant	12 days	<1
HFO-1234ze, (*E*)-HFC=CHCF_3_	Refrigerant	10 days	<1
HFO-1336-mzz, (*Z*)-F_3_CCH=CHCF_3_	High temperature refrigerant	27 days	2
SF_6_	Insulation gas in electric power industry	3200	25 200
PFC-14, CF_4_	Semiconductor manufacture, aluminium production	50 000	7380
PFC-116, C_2_F_6_	Semiconductor manufacture	10 000	12 400
PFC-218, C_3_F_8_	Semiconductor manufacture	2600	9290

### 3.1 HCFCs and HFCs

Trifluoromethane, CF_3_H (HFC-23), is produced on a vast scale (*ca.* 20 Gg year^−1^) largely as a by-product in the synthesis of CF_2_ClH (HCFC-22) by the over-fluorination of chloroform (CCl_3_H).^60^ HFC-23 is a potent greenhouse gas, with a GWP_100_ of 14 600 and an atmospheric lifetime of 228 years.^59^ It has very little application itself and is either stored, destroyed or released to the environment. Its atmospheric release therefore presents a significant issue for climate change. Although commercial applications of HCFC-22 are being phased out under the Montreal Protocol, HCFC-22 is still permitted for ‘non-dispersive uses’, such as a chemical intermediate in the manufacture of fluoropolymers.^20^ Total HCFC-22 production has increased dramatically, from 65 Gg year^−1^ in 1990 to 947 Gg year^−1^ in 2017.^61^ In more recent years, this production has been dominated by developing countries, but there are still small increases in total production from developed countries despite a complete phase-out of production and consumption scheduled for 2030.^61^ As a consequence of increased production of HCFC-22, the atmospheric concentration of HFC-23 has been steadily increasing since at least 1978.^62,63^ Using data collected from AGAGE measurements, global emissions of HFC-23 were estimated as ∼15.9 Gg year^−1^ in 2018, the highest than at any point in history,^61^ an increase from the estimates of ∼12.3 Gg year^−1^ in 2016, ∼12.5 Gg year^−1^ in 2012, ∼8.9 Gg year^−1^ in 1995 and ∼4.3 Gg year^−1^ in 1978.^18,64^ It is noted that there was brief mitigation of global HFC-23 emissions during 2006–2009, due to initial success of abatement technologies through the UNFCCC Clean Development Mechanism (CDM), with emissions reaching a global minimum of ∼9.6 Gg year^−1^ in 2009.^18,20^ It is suggested that abatement measures have since been inadequate to offset the increasing production of HCFC-22 for non-dispersive uses, hence the return to an upwards trend in global emissions of HFC-23.^20^ Estimates of global emissions of HCFC-22 have remained relatively constant at ∼370 Gg year^−1^ during the period 2012–2016.^56^ Before this period of stability they increased drastically to ∼368.8 Gg year^−1^ in 2012, from ∼232.2 Gg year^−1^ in 1995 and ∼101.8 Gg year^−1^ in 1978.^18^

Production and emissions of 1,1,1,2-tetrafluoroethane (HFC-134a) begun in the 1990s as CFCs were being phased-out. HFC-134a was seen as a direct replacement for CFC-12 in motor vehicle air conditioning systems.^17^ HFC-134a is manufactured predominantly by fluorination of trichloroethane using hydrogen fluoride in the gas phase, with a heterogenous catalyst. The trichloroethane is itself synthesised from ethylene.^23^ HFC-134a has a GWP_100_ of 1530 and an atmospheric lifetime of 14 years.^59^ It is the most abundant HFC in the global atmosphere.^65^ Estimates suggest that more than 56% of cumulative HFC-134a produced since 1990 has been released to the environment.^19^ Global emission estimates derived from HFC-134a atmospheric concentration data collected from AGAGE measurements suggest a dramatic increase to ∼223 Gg year^−1^ in 2016, from ∼176.5 Gg year^−1^ in 2012, ∼109.6 Gg year^−1^ in 2003 and ∼10.2 Gg year^−1^ in 1994.^18,64^

1,1,1-Trifluoroethane (HFC-143a) is used in a variety of refrigerant blends, mainly R-404A.^29,34,36,66,67^ It was avoided as a pure refrigerant due to its high GWP_100_ of 5810 and because it is also considered mildly flammable.^22,34,59,68^ Recent research has looked at the critical properties of a binary mixture of HFC-143a and *E*-HFO-1234ze as an alternative refrigerant and supercritical solvent, due to the low critical temperature and high chemical stability of HFC-143a.^34^ The global emission estimates of HFC-143a have shown an increase from ∼1 Gg year^−1^ in the early 1990s to ∼23 Gg year^−1^ in 2012 and to ∼28 Gg year^−1^ in 2016.^18,64^

1,1-Difluoroethane (HFC-152a) has a relatively low GWP_100_ of 164 and was initially considered as a replacement refrigerant for CFC-12.^59,69–74^ However, unlike HFC-134a it is flammable, and as a result it is limited to usage as a component of refrigerant blends and as a foam blowing agent or propellant in aerosol sprays and gas dusters.^75^ The global emission estimates of HFC-152a have shown an increase from ∼2 Gg year^−1^ around 1990, to ∼53 Gg year^−1^ around 2012 and remained at ∼53 Gg year^−1^ in 2016.^18,64^

Difluoromethane (HFC-32) has a relatively low GWP_100_ of 771 and good thermodynamic properties as a refrigerant. Its usage as a pure refrigerant was limited due to the fact that it is marginally flammable.^19,59,76^ Similarly, pentafluoroethane (HFC-125) was considered another viable candidate for refrigeration, but it has a relatively high GWP_100_ of 3740.^59,68,77,78^ These gases are often used in refrigerant blends, such as HFC-410A (1 : 1, HFC-32 : HFC-125).^19^ This blend is a primary replacement for HCFC-22 and thus HFC-410A is now widely used in residential and commercial air conditioning. The blend has practically zero ozone-depleting potential (ODP), in this sense superior to HCFC-22, however the GWP_100_ of HFC-410A is a 16% increase from HCFC-22. This provides an example of an environmental trade-off between ozone depletion and greenhouse gas emissions. HFC-125 is also used as fire suppression agents, owing to its electrical non-conductivity, ready vapourisation, low toxicity and non-flammability.^22^ The global emission estimates of HFC-125 have grown from ∼1 Gg year^−1^ in the early 1990s to ∼40 Gg year^−1^ in 2012 and to ∼62 Gg year^−1^ in 2016, while HFC-32 global emissions have increased from ∼1 Gg year^−1^ in the early 2000s to ∼20 Gg year^−1^ in 2012 and to ∼35 Gg year^−1^ in 2016.^18,64^

### 3.2 Perfluorinated F-gases

SF_6_ is considered one of the most potent greenhouse gases, with a GWP_100_ of 25 200 and an atmospheric lifetime of 3200 years.^38,59,79–81^ SF_6_ is largely immune to chemical and photolytic degradation, evidenced from vertical profiles of SF_6_ indicating very little loss of SF_6_ due to photochemistry in the troposphere and lower atmosphere,^38,82^ meaning that its contribution to global warming is cumulative and effectively permanent.^40,83^

The world production of SF_6_ has steadily increased since the 1970s. The large amount of SF_6_ in electrical equipment provides a substantial source of emissions through maintenance, replacement, and leakage. It is estimated that about 12% of SF_6_ consumed in the manufacture and commissioning of electrical equipment is directly emitted.^38^ In the UK it is estimated that SF_6_ losses from the electrical power industry is an average of 1% per year from 2010–2016.^38,84^ The global atmospheric emission rate of SF_6_ was estimated as ∼9.0 Gg year^−1^ in 2018, an increase from ∼7.3 Gg year^−1^ in 2008, and from 2.5 Gg year^−1^ in 1978, using atmospheric concentration data collected at the AGAGE monitoring sites.^38^ The overall increase is consistent with the increase in globally installed electrical capacity.^38^ More recent increases are attributed to the rapid expansion of the electric power industry particularly in Asia.^38^ For example, electrical capacity installed in China relative to the rest of the world increased from ∼3% in 1980 to ∼43% in 2018.^38,85^ The adoption of renewable energy technologies has been particularly important.^85^ It has been assumed that renewables have a wider distribution of power (compared to localised gas or oil power stations), which consequently requires more connections in the electricity grid and therefore more gas-insulated switches, circuit breakers and transformers.^38^ As a result, despite an observed decrease in SF_6_ emissions from developed countries in recent years thanks to efforts made to replace SF_6_ as a blanketing gas along with technological improvements to reduce emissions from electrical equipment, this has been largely overwhelmed by major increases in emissions elsewhere in the world.^38^

Perfluorocarbons such as CF_4_ (PFC-14), C_2_F_6_ (PFC-116) and C_3_F_8_ (PFC-218) are amongst the most highly potent greenhouse gases regulated under the Kyoto Protocol.^47^ CF_4_ has a GWP_100_ of 7380 and an atmospheric lifetime of 50 000 years, C_2_F_6_ has a GWP_100_ of 12 400 and an atmospheric lifetime of 10 000 years, and C_3_F_8_ has a GWP_100_ of 9290 and an atmospheric lifetime of 2600 years.^59^ These gases are vital to the semiconductor industry for numerous low-pressure operations such as chemical etching. The gaseous waste streams from these processes are often emitted to the atmosphere due to the difficulty of thermal incineration processes. Significant CF_4_ releases also occur during the electrolytic production of aluminium.^47^ CF_4_ and C_2_F_6_ were found to be ubiquitous in the troposphere from the 1970s, with the less abundant C_3_F_8_ being found in the atmosphere more recently.^47^ It is noted that there is evidence for a natural source of CF_4_ in the atmosphere, likely from a lithospheric source, although there is some uncertainty in its contribution to emissions.^47^ Reports using atmospheric concentration data collected by AGAGE estimated the global CF_4_ emissions to be ∼13.9 Gg year^−1^ in 2019, ∼12.6 Gg year^−1^ in 2016 and ∼10.5 Gg year^−1^ during the period 2005 to 2008.^47,55,56^ This is slightly down from earlier emission rates, for example ∼17.7 Gg year^−1^ from 1980–1984.^47^ The reduction is attributed to increasing efficiency and reduction measures taken up by the aluminium industry. The estimated global emissions of C_2_F_6_ showed an increase from ∼2.0 Gg year^−1^ for 1980–1984 to ∼2.9 Gg year^−1^ during 2000–2004, before decreasing slightly to ∼2.3 Gg year^−1^ in 2005–2008. Global emissions of C_2_F_6_ were then estimated as ∼2.0 Gg year^−1^ in 2016 and ∼2.2 Gg year^−1^ in 2019.^47,55,56^ This trend follows the introduction of C_2_F_6_ as a fluorine source in the semiconductor industry in the early 1990s, before its gradual replacement with more efficient fluorine sources such as NF_3_ in more recent years.^47^ C_3_F_8_ global emissions increased sharply to ∼1.1 Gg year^−1^ in 2000–2004 from ∼0.2 Gg year^−1^ in 1980–1984, before declining to ∼0.8 Gg year^−1^ for 2005–2008 and ∼0.5 Gg year^−1^ for 2016–2019.^47,55,56^ This trend represents the increased applications for C_3_F_8_ in more recent years, such as an inert reaction medium, a dielectric and as a propellant, before attempts to gradually replace it.^47^ While the overall emissions of these gases are lower compared to CFCs, HCFCs and HFCs discussed above, their incredibly long atmospheric lifetimes can lead to permanent alteration of the radiative budget of the atmosphere and so even small atmospheric concentrations are to be taken seriously.^47^

**Fig. 2:**
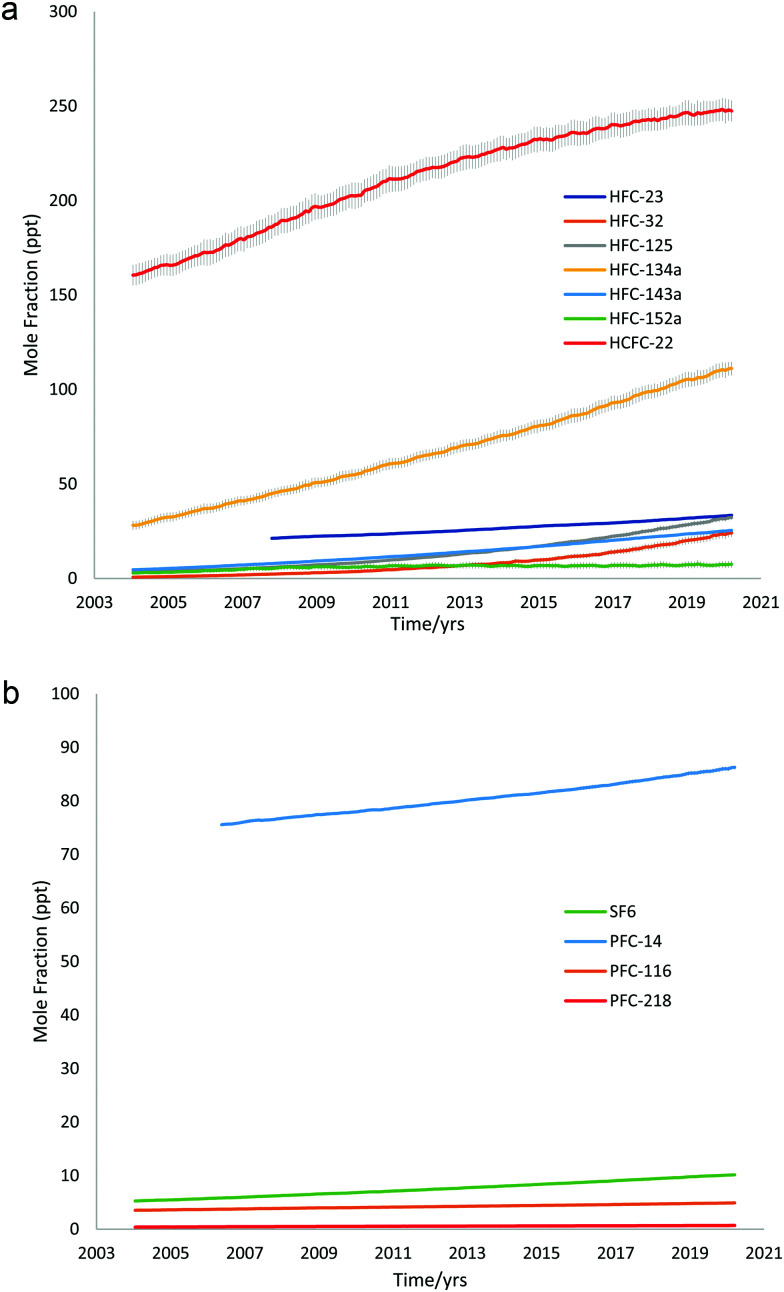
(a) Global mean atmospheric concentrations of HFCs and HCFC-22 *vs.* time, data from AGAGE GC-MS Medusa measurements. (b) Global mean atmospheric concentrations of PFCs and SF_6_*vs.* time, data from AGAGE GC-MS Medusa measurements.

Fig. 2a and b display the global mean atmospheric concentrations of the above synthetic greenhouse gases, showing their variation against time. Data was obtained from AGAGE GC-MS “Medusa” measurements, including the most recent data published (2020) at the time of writing.^51^

## 4. Destruction of F-gases

One of the ways to prevent the release of environmentally persistent F-gases into the atmosphere is through collection and immediate destruction of the emitted gases. Destruction methods for F-gases require extreme conditions due to their chemical and physical inertness. They are as a result often highly energy intensive, with methods including thermal oxidation, catalytic hydrolysis and plasma destruction.^62^

### 4.1 Thermal oxidation

One of the most common destruction methods is thermal oxidation. The thermal oxidation of HFC-23 (which often contains traces of HCFC-22) generally requires temperatures higher than 1200 °C and leads to the formation of CO_2_, HF and HCl. The HF can be recovered and sent to storage for future use, often as an alkali fluoride salt, and as such this destruction technique can allow for some fluorine material recovery. A major issue of this technique is the high operational costs associated with sustaining these extremely high temperatures and finding suitable materials compatible with both the temperature and the strongly corrosive HF. Moreover, there is the potential to form other hazardous materials during the cooling procedure of reaction flows that may pose a great environmental threat, such as dioxin.^62^ Under the UNFCCC Clean Development Mechanism (CDM) (2003–2014), developing countries were able to register waste treatment facilities for thermal oxidation of synthetic greenhouse gases (not including CO_2_).^61^ There were 19 HCFC-22 production plants in five developing countries approved for the destruction of waste HFC-23.^20,62^ As noted in Chapter 3, global emissions of HFC-23 declined between 2006–2009, and this is largely attributed to CDM projects. Since the CDM period ended, HFC-23 emissions have grown once more.^20,61^

A few studies available in the open literature have focused specifically on HFC-134a destruction.^86–91^ One study carried out experiments using a tubular-type furnace to explore the important features of HFC-134a thermal decomposition. They concluded that with a temperature above 800 °C, sufficient O_2_ and an auxiliary fuel (H_2_O or CH_4_), HFC-134a reacts to produce HF, H_2_O and CO_2_.^86^ Without an auxiliary fuel capable of providing a hydrogen atom, HFC-134a reacts with O_2_ to produce F_2_ instead of HF. F_2_ is highly toxic and far more difficult to remove than HF.^86^ With insufficient H_2_O and O_2_, carbon products such as CO can be formed.^87^ A fine control over other factors such as steam flow rate are crucial for keeping the NO_*x*_ concentration down. It appears paramount that the conditions are optimum for a destruction methodology to be both environmentally and economically viable. There are also a few examples of patents filed for the thermal oxidation of other F-Gases, such as SF_6_ and other PFCs.^92,93^ For SF_6_, temperatures in excess of 1100 °C are required for its combustion, and even at these temperatures combustion is often incomplete with NO_*x*_ released, along with the same problems of cost and sourcing of materials.^81^

### 4.2 Catalytic hydrolysis

Catalytic hydrolysis has been thoroughly researched as an alternative, low-cost method for F-gas destruction. For this approach to be viable, catalytic materials must be both highly reactive and be able to function for long periods of time in a highly corrosive acid gas environment.^94^ Multiple reports, along with a number of patents,^95–97^ have demonstrated successful catalyst systems for the disposal of HFC-23 alongside other HCFCs, CFCs and PFCs, but only at very low concentrations. For example, a Pt/ZrO_2_–SO_4_ catalyst was shown to destroy HFC-23 by catalytic hydrolysis to form CO_2_ and HF, at 500–550 °C.^94^ In a similar manner, a nickel pyrophosphate catalyst was shown to decompose HFC-23 at 500 °C to CO_2_ and HF.^98^ Another report detailed a AlPO_4_–Al_2_O_3_ system that was shown to hydrolytically decompose CF_4_ into CO_2_ and HF, where the phosphate helped stabilise the catalyst from deactivation.^99^ Zhang and co-workers reported a platinum promoted TiO_2_–ZrO_2_ catalyst that served to convert HCFC-22 to CO_2_ with 95% selectivity at 340 °C.^100^ A major downside to these methods is that at high concentrations (>5000 ppm), the efficiency and stability of the catalysts are highly vulnerable.^62^ Also, the inclusion of an expensive transition metal is often required for the catalyst to convert CO to CO_2_ during hydrolysis and also allows for a lower operating temperature.^62,99^ The production of HF and HCl pose a huge challenge to any catalyst, as these compounds will react with almost all materials leading to catalyst deactivation. Catalytic technologies for SF_6_ destruction suffer in a similar way as the toxic products of decomposition poison the catalyst.^101^

### 4.3 Plasma destruction

Plasma technology has been commercially applied for the destruction of F-gases in a plasma arc at temperatures typically between 10 000 and 30 000 °C.^62,102–106^ The PLASCON process technology, developed by the Australian Commonwealth Scientific and Research Organisation and SRL Plasma Ltd, was adapted for CFC and halon destruction in 1992, and for HFC-23 in 2007. The fluorinated products of HFC-23 destruction are HF and F_2_. These molecules create significant issues at the high temperatures required. In the late 1990s, a few reports detailed the abatement of HFC-23 and PFCs using surface wave plasma with very high efficiency, but poisonous COF_2_ was detected in the effluent gas alongside other products including CO, CO_2_ and HF.^62,107,108^ Methods to avoid the formation of other dangerous fluorochemicals have been explored, such as by using steam instead of oxygen as the oxidising gas.^104^ Nonthermal plasma technologies have been thoroughly investigated as a way of controlling PFCs, although these also often led to the formation of toxic byproducts.^107–112^ Combining plasma with a catalyst was explored in the abatement of C_2_F_6_, where it was found to produce mainly CO_2_ and trace CO, as the catalyst surfaces were able to adsorb and dissociate radicals that usually lead to the formation of toxic products such as F_2_, COF_2_ and CF_4_.^113^ In general, the potential for hazardous fluorinated by-products along with the operational costs associated with the exceptionally high temperatures required restrict the wide application of thermal plasma technology for F-gas destruction.^62^

### 4.4 Electric discharge

SF_6_ can undergo decomposition in an electrical discharge (arc, spark or corona).^48,113–122^ However, the products from this type of decomposition can be highly toxic, while some are potent greenhouse gases themselves. Decomposition products can include S_2_OF_10_, CF_4_, COF_2_, F_2_, HF, H_2_S, NF_3_, F_2_O, SiF_4_, SO_2_, S_2_F_10_, SF_4_, SO_2_F_2_, SOF_2_, SOF_4_, S_2_O_2_F_10_.^48,114,118,123–125^

## 5. Chemical upgrading of F-gases

Much progress has been made in the field of chemical upgrading fluorocarbons into reactive building blocks, but F-gases are some of the most challenging and least reactive molecules to be studied.^126–129^ This is largely due to the strength of the sp^3^ C–F bond; the C–F bond dissociation energy of CH_3_F is 115 ± 4 kcal mol^−1^.^130^ In addition, alkyl fluorides are poor substrates for nucleophilic substitution or oxidative addition to metals due to a lack of charge stabilisation in the transition state for bond-breaking.^130–132^ Despite this, recent work has shown a range of methods for C–F activation in fluoroalkanes.^133–136^ We describe below the recent efforts in targeting new reactions to upgrade F-gases.

### 5.1 HFC-23

Fluorination in the pharmaceutical and agrochemical industries is increasingly important, with both –CF_3_ and –CF_2_H moieties common in drug design. Therein lies an opportunity for a small component of waste CF_3_H (HFC-23) to serve as a feedstock for the synthesis of trifluoromethyl and difluoromethyl building blocks. CF_3_H has a low boiling point (−83 °C) and a relatively acidic C–H bond (p*K*_A_ ∼ 25 in H_2_O).^60,137^ The CF_3_^−^ anion generated from a deprotonation reaction has been shown to decompose under certain reaction conditions to form difluorocarbene, CF_2_, and a fluoride anion, F^−^.^60,138–142^

#### Trifluoromethylation

**Scheme 1:**
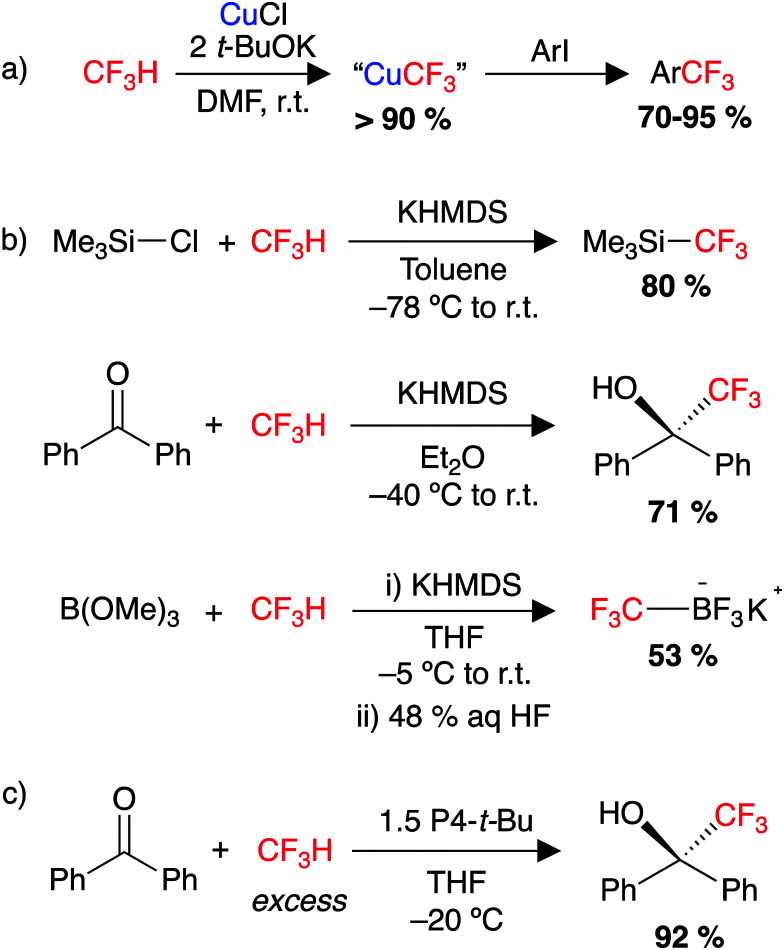
(a) Cupration of CF_3_H and trifluoromethylation of aryl iodides, (b) nucleophilic trifluoromethylation of a range of electrophiles using CF_3_H as a feedstock. HMDS = hexamethyldisilazane, (c) trifluoromethylation of carbonyl substrates (benzophenone shown) using HCF_3_ and P4-*t*-Bu superbase. P4-*t*-Bu = ((NMe_2_)_3_P=N)_3_P=N(*t*-Bu).

Much of the work with CF_3_H has focussed on deprotonation and subsequent transfer of the CF_3_^−^ anion to an electrophile. Early work relied on using a strong base along with DMF to stabilise the CF_3_^−^ anion,^143–146^ or by using electrochemically generated bases.^147,148^ Grushin and co-workers developed a method to cuprate CF_3_H to form “CuCF_3_” derivatives, which were shown to be capable of trifluoromethylating organic electrophiles such as haloarenes and haloheteroarenes, aryl boronic acids and α-haloketones (Scheme 1a).^149–151^ A major breakthrough came in 2012 when Prakash and co-workers reported a method for nucleophilic trifluoromethylation of a range of silicon-, boron-, sulfur- and carbon-based electrophiles using CF_3_H as a feedstock (Scheme 1b).^60^ One product described by this approach is Me_3_SiCF_3_, the Ruppert–Prakash reagent widely used as for installation of CF_3_ groups.^152–160^ In 2013, Shibata and co-workers utilised the steric bulk of a P4-*t*-Bu superbase to form a stable CF_3_^−^ anion from HCF_3_, which can trifluoromethylate carbonyl compounds (Scheme 1c).^161^ They were later able to demonstrate this reaction using a catalytic amount of the superbase,^162^ along with an extension of the substrate scope.^162–164^ They also reported the use of potassium bases in the presence of a polyether solvent such as triglyme for the trifluoromethylation of a range of carbonyl compounds using HCF_3_.^165,166^ For enantioenriched substrates a stereodivergent trifluoromethylation was reported, depending on the base used.^163,164^ A more recent strategy utilised borazine as a Lewis acid to form highly reactive CF_3_^−^ adducts from a reaction of CF_3_H with an alkali metal hydride. The CF_3_^−^ adducts were then shown to transfer CF_3_^−^ to a wide range of electrophiles with quantitative regeneration of free borazine.^167,168^ There is limited transition metal chemistry with CF_3_H. In 2011, a report detailed the oxidative addition of the C–H bond of CF_3_H by an Ir(i) complex, the product of this reaction was only observed by NMR spectroscopy at −10 °C.^169^

#### Difluoromethylation

**Scheme 2:**
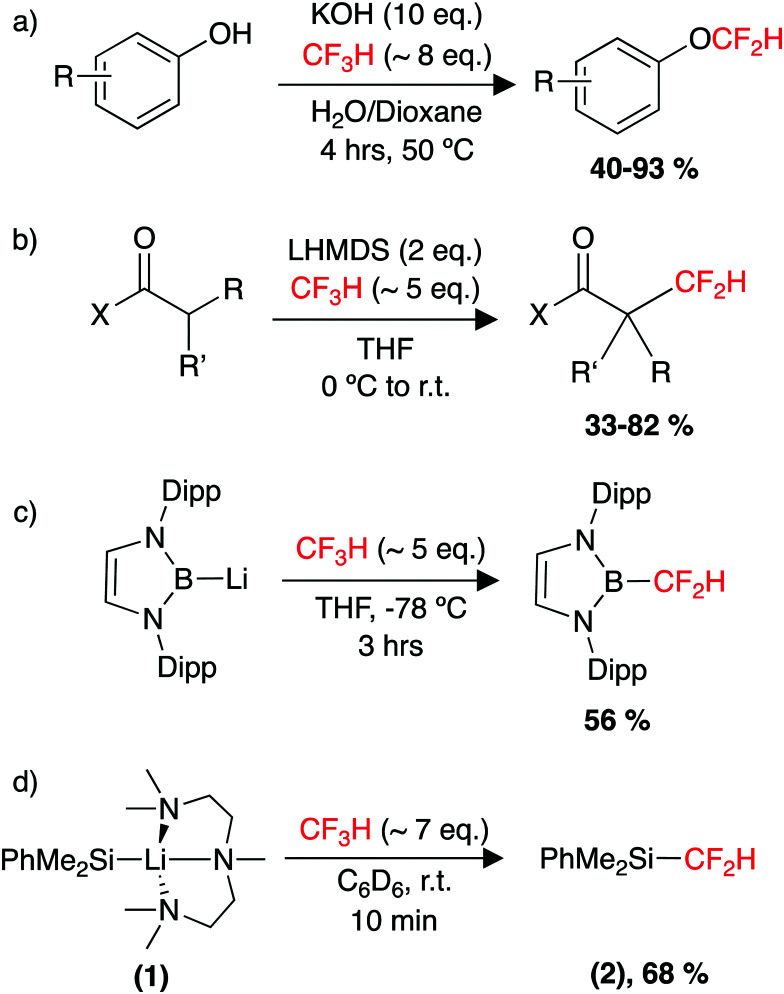
Utility of HCF_3_ as a feedstock, (a) in the synthesis of aryldifluoromethylethers, (b) in the α-difluoromethylation of carbonyls, (c) defluoroborylation to form an organoboron building block and (d) defluorosilylation to form an organosilicon building block.

Despite receiving less attention than trifluoromethylation, CF_3_H has also been targeted as a {CF_2_H}^+^ synthon, *via* functionalisation of the C–F bond. In 2013, Dolbier Jr. and co-workers reported a method to difluoromethylate a range of phenols and aromatic thiols using CF_3_H (Scheme 2a).^170^ The reaction required a large excess of CF_3_H and use of a strong base (KOH), which limited functional group tolerance. Nevertheless, this represented a promising new method for the synthesis of simple aryldifluoromethyl ethers, which had often relied on the ozone depleting CF_2_ClH. In 2012, the Mikami group demonstrated a method for the α-difluoromethylation of carbonyl substrates using LiHMDS and an excess of CF_3_H.^171^ The scope included a range of cyclic and acyclic esters and amides (Scheme 2b). Interestingly, lithium was the only alkali metal capable of achieving the C–F activation. This has been attributed to stronger Li⋯F interactions compared to those of Na⋯F and K⋯F (Δ*H*_lattice_ = 251, 222 and 198 kcal mol^−1^, respectively).^172,173^ Trapping experiments showed no evidence for a carbene mechanism, while a follow up computational study proposed a S_N_2-type pathway for C–F cleavage after initial deprotonation of CF_3_H.^174^ The group later extended their methodology for the difluoromethylation of acidic sites of terminal alkynes and nitrile compounds,^175,176^ while at a similar time the Shibata group reported a system for difluoromethylating terminal alkynes using HCF_3_.^177^

More recently in 2017, the groups of Ito and Mikami reported the defluoroborylation of CF_3_H using a highly nucleophilic boryl lithium reagent, to form a –CF_2_H containing organoboron building block (Scheme 2c).^178^ While a mechanistic study was not carried out for this system, the related computational study by Mikami on the α-difluoromethylation of lithium enolates was utilised to propose a pathway for the defluoroborylation.^174^ The authors suggest initial deprotonation of CF_3_H occurs to form the unstable intermediate [CF_3_Li], before C–F cleavage then proceeds *via* an S_N_2-type attack by the nucleophilic boryl lithium at [CF_3_Li], in a bimetallic transition state. While an important discovery, application of the defluoroborylation methodology was limited by issues regarding scalability. The boryl lithium reagent is extremely difficult to synthesise and is highly susceptible to degradation. The difluoromethyl organoboron building block was reported as bench stable but its utility is unknown.^178^

**Fig. 3:**
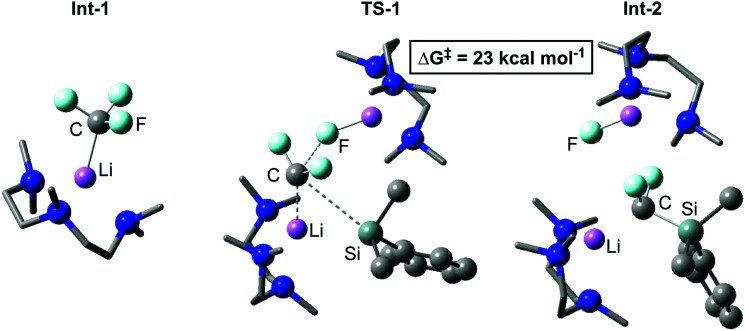
Key stationary points calculated for the C–F cleavage of an equivalent of [CF_3_Li·PMDETA] in the defluorosilylation of trifluoromethane.

In 2020, our group reported the rapid, room-temperature defluorosilylation of CF_3_H with the silyl lithium reagent 1 (Scheme 2d).^179^ The reaction forms a valuable difluoromethyl organosilicon building block, 2, which is closely related to Me_3_Si–CF_2_H, both are known difluoromethyl transfer agents.^180,181^ The reaction was successfully scaled up to synthesise 2 on a gram-scale in a 68% isolated yield. An extensive DFT study was carried out to explore the mechanism of the reaction. The mechanism is proposed to involve an initial deprotonation of CF_3_H by 1 to form PhMe_2_SiH and [CF_3_Li·PMDETA] (PMDETA = *N*,*N*,*N*′,*N*′′,*N*′′-pentamethyldiethylenetriamine), a second equivalent of 1 then attacks [CF_3_Li·PMDETA] at the carbenoid carbon in an S_N_2-like fashion, resulting in the cleavage of a C–F bond and formation of a C–Si bond. Reprotonation by a further equivalent of CF_3_H leads to the product 2 and regenerates an equivalent of [CF_3_Li·PMDETA], suggesting the reaction is catalytic in [CF_3_Li·PMDETA]. Key intermediates and a transition state of the calculated pathway are represented in Fig. 3.

TS-1 is unusual as [CF_3_Li] may have been considered an unlikely electrophile. A Natural Bond Orbital (NBO) analysis was therefore carried out. The resulting Natural Population Analysis (NPA) charges revealed a positive charge at the carbenoid carbon of [CF_3_Li·PMDETA] as a result of the highly electron withdrawing effect of three electronegative fluorine atoms, a result noted in previous calculations on [CF_3_Li].^182^ This explains the electrophilic nature of [CF_3_Li·PMDETA] and why it is attacked by 1 in TS-1. The geometry of TS-1 is somewhat similar to that calculated by Mikami for the attack of a THF-stabilised lithium enolate on [CF_3_Li].^174^TS-1 is bimetallic, where one lithium cation stabilises the fluoride leaving group, and the other acts as an anchor in C–Si bond formation. It has been suggested in other work that Li⋯F interactions are crucial in stabilising similar transition states.^171,174^

**Scheme 3:**
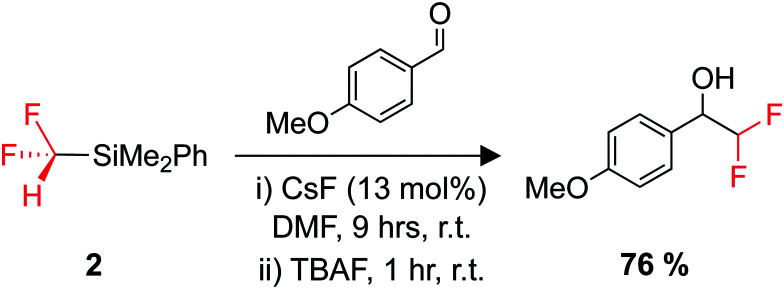
Utility of 2 as a difluoromethylating agent. TBAF = tetrabutylammonium fluoride.

The organosilicon building block 2 is an easy-to-use difluoromethyl transfer agent for carbonyl substrates (Scheme 3).^180,181^ The use of 2 has been somewhat scarce compared to Me_3_SiCF_2_H, but it is suggested that this could be due to the difficulty and cost of its synthesis.^183^ Greater access to easy-to-use fluorinated transfer agents often leads to an increased use of those fluorinated moieties in pharmaceutical design.^179^

#### Flow chemistry

The potential for scalable methods to utilise CF_3_H has been demonstrated using flow chemistry.^184–186^ Kappe and coworkers successfully demonstrated the difluoromethylation of diphenylacetonitrile with CF_3_H in flow,^187^ adopting the conditions published by Mikami.^175^ They have also demonstrated the synthesis of α-difluoromethyl amino acids using CF_3_H in a continuous flow process.^188^ Finally, in 2018 the same group reported the continuous flow synthesis of eflornithine, an active pharmaceutical ingredient listed on the World Health Organisation's essential medicines, using CF_3_H as the feedstock.^189^ The reaction produced 19.5 g of the active agent, in an 86% yield, requiring only 1.05 equivalents of CF_3_H, with a reaction time of less than 25 minutes. In 2019, Shibata and coworkers reported a strategy for trifluoromethylating carbonyl compounds using HCF_3_ in flow.^184^ A recent review article discusses strategies for using CF_3_H and other fluorinated greenhouse gases in flow.^13^

### 5.2 HFC-134a

**Scheme 4:**
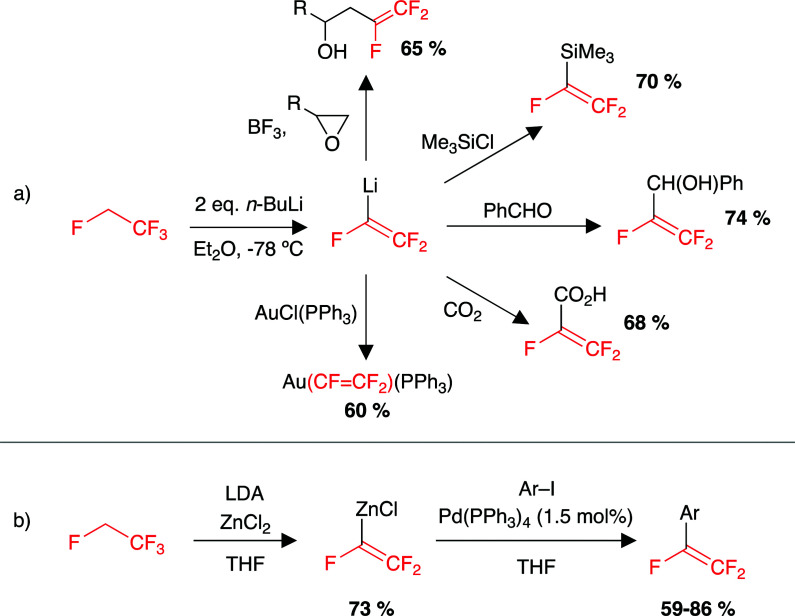
Examples of HFC-134a as a source of the trifluorovinyl moiety and utility in (a) reactions with a range of electrophiles, (b) palladium catalysed cross-coupling reactions. LDA = lithium diisopropylamide.

The chemistry of HFC-134a (1,1,1,2-tetrafluoroethane) is largely limited to reactions with a base that involve a formal elimination of an equivalent of HF and subsequent deprotonation to form a nucleophilic source of the trifluorovinyl moiety.^190,191^ For example, the reaction of HFC-134a with 2 equiv. *n*-BuLi at −78 °C formed trifluorovinyllithium, reported to be stable in solution at −78 °C (Scheme 4a). Warming the solution to room temperature led to decomposition to an uncharacterised black material. The trifluorovinyllithium moiety formed at −78 °C can react with a wide range of electrophiles including metal halides, main group halides, CO_2_, aldehydes and epoxides.^190–208^ Using this method with transition metal halides resulted in the formation of transition metal complexes with a trifluorovinyl ligand.^191,193^ Other reports include the transfer of the trifluorovinyl group onto zinc chlorides for application in palladium-catalysed Negishi cross-coupling reactions (Scheme 4b).^201,209–213^

There is a distinct lack of sp^3^ C–F functionalisation chemistry of HFC-134a. This is likely due to the high bond strength of the sp^3^ C–F bond, and as the literature suggests, deprotonation and elimination to form trifluorovinyl species appears to be far more facile.

### 5.3 HFC-143a, HFC-152a, HFC-32 and HFC-125

**Scheme 5:**
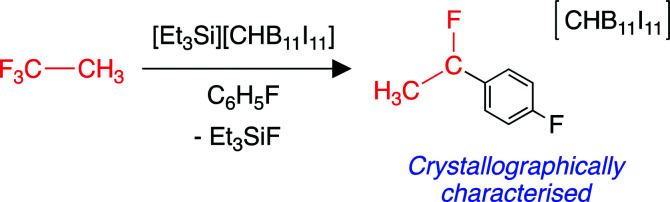
Fluoride abstraction from CF_3_CH_3_ by a main group cationic species.

A highly fluorophilic main group cation with a carborane counterion has been reported to abstract fluoride from CF_3_CH_3_ (HFC-143a).^214^ The putative CH_3_CF_2_^+^ carbocation generated from this reaction can undergo a Friedel–Crafts addition to the fluorobenzene solvent, to form (*p*-F–C_6_H_4_)(CH_3_)CF^+^, which was isolated as a salt with the carborane anion (Scheme 5). The encapsulation of halocarbons (including CF_3_CH_3_) in caged supramolecular systems has also been reported.^215^ In general, there is a lack of functionalisation chemistry for CF_3_CH_3_, likely due to the very strong C–F bonds possessed by a terminal CF_3_ group. There is a similar lack of activation chemistry found in the literature of CHF_2_CH_3_ (HFC-152a).

**Scheme 6:**
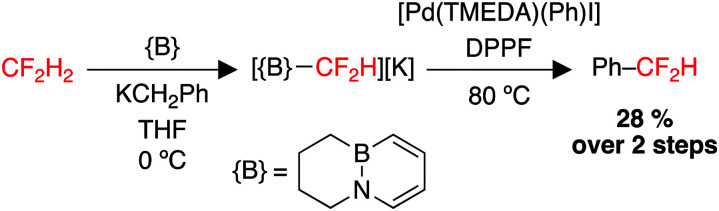
Utility of H_2_CF_2_ as a {CF_2_H}^−^ building block. DPPF = 1,1′-bis(diphenylphosphino)ferrocene. TMEDA = *N*,*N*,*N*′,*N*′-tetramethylethylenediamine.

The use of CF_2_H_2_ (HFC-32) a nucleophilic source of the difluoromethyl group would be highly attractive, but has been largely limited by the weak acidity of CF_2_H_2_ (p*K*_A_ = 35–41, gas phase proton affinity: 389 kcal mol^−1^) and low stability of the conjugate base to α-fluoride elimination.^216,217^ An elegant approach published in 2019 demonstrated the first strategy to repurpose difluoromethane as a {CF_2_H}^−^ building block, through the use of a Lewis acid/base pair to deprotonate CF_2_H_2_ and capture CF_2_H^−^ as a borane adduct. This adduct can then serve as a nucleophilic source of CF_2_H^−^, capable of transmetallation to Pd(ii) and subsequent reductive elimination to form a C–C coupled product (Scheme 6).^218^

Hydrodefluorination of CF_2_H_2_ has also been reported. In 2001, Jones and co-workers reported the reaction of a range of fluorocarbons with [Cp*_2_ZrH_2_] (Cp* = pentamethylcyclopentadienyl). The hydrodefluorination of CF_2_H_2_ required heating to 120 °C for more than 10 days, giving methane and [Cp*_2_ZrHF]. The authors proposed a radical chain mechanism.^219^ Andersen and coworkers showed that CF_2_H_2_ reacted with a cerium hydride compound to form the analogous cerium fluoride and CH_3_F.^220^ The oxidative addition of the C–H bond of CF_2_H_2_ to a rhodium compound under photolytic conditions has been reported, where the product was then shown to reductively eliminate CF_2_H_2_ in C_6_D_6_ at 100 °C.^221^ In 2006, a paper disclosed the gas-phase C–F activation of CF_2_H_2_ by laser-ablated Ti atoms to form the methylidene complex [CH_2_=TiF_2_],^222^ and later with other transition metals.^223–227^ More recently, C–F activation of difluoromethane was reported using heterogeneous nanoscopic aluminium chlorofluoride in the presence of Et_3_SiH, forming Et_3_SiF amongst other products.^228^ A surface-bound silylium-like ion was proposed to be a crucial intermediate for C–F cleavage.

**Scheme 7:**
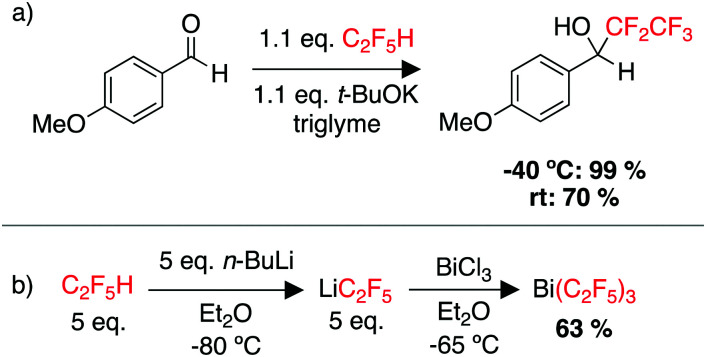
Examples of using C_2_F_5_H as a feedstock for (a) pentafluoroethylation of a carbonyl substrate, (b) the synthesis of a main group complex with C_2_F_5_ ligands.

While there remains a lack of C–F functionalisation chemistry for C_2_F_5_H (HFC-125), there are quite a few examples of using pentafluoroethane as a source for installing the {C_2_F_5_} moiety onto a range of electrophiles, through deprotonation and trapping of [C_2_F_5_]^−^.^229–234^ In one example, Shibata and coworkers used a potassium base in combination with triglyme to encapsulate the K cation. The pentafluoroethyl moiety could then be transferred to a wide range of carbonyl substrates (Scheme 7a).^234^ A recent report demonstrated flow-conditions for the pentafluoroethylation of a wide range of ketones, aldehydes and chalcones.^235^ There has also been work on the cupration of C_2_F_5_H and the use of “CuCF_2_CF_3_” as a pentafluoroethylation reagent.^236,237^ Tsui and co-workers reported “CuCF_2_CF_3_” as a source of the ˙CF_2_CF_3_ radical for pentafluoroethylation of unactivated alkenes.^238^ Perfluoroalkyl groups can be used to increase the Lewis acidity of main group compounds, and thus C_2_F_5_H as a source of LiCF_2_CF_3_ has been employed in the synthesis of new main group species such as Bi(C_2_F_5_)_3_ (Scheme 7b).^239–243^ It is also utilised in ligand design for transition metal complexes.^231^

### 5.4 SF_6_

**Scheme 8:**
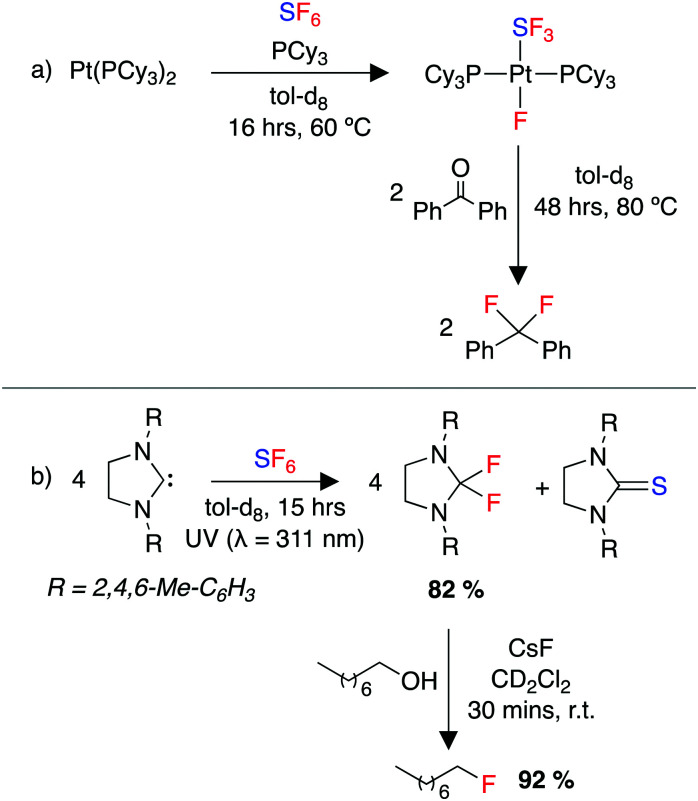
(a) Activation of SF_6_ by a Pt(0) complex and use of the product in a subsequent deoxyfluorination of a ketone, (b) photolytic activation of SF_6_ using an NHC, and subsequent deoxyfluorination of an alcohol.

Early work in the field of SF_6_ degradation described the chemical breakdown of SF_6_ using strong reducing agents such as alkali metals.^244–247^ More recently, the field has been reinvigorated by multiple reports of SF_6_ activation using transition metals.^248–255^ Stoichiometric reactions of low valent, early transition-metal compounds of Ti, Zr, Cr and V with SF_6_ produced a range of transition metal fluorides.^248,249^ A β-diketiminate nickel complex was shown to activate SF_6_, forming nickel(ii) fluoride and nickel(ii) sulfide products. Braun and co-workers reported the selective decomposition of SF_6_ using rhodium complexes in both stoichiometric and catalytic transformations. These reactions include a range of fluorine and sulfur scavengers to give a range of fluoride and sulfide decomposition products.^251,252^ The same group later reported the activation of SF_6_ by a xantphos ligated rhodium complex to form well defined rhodium fluoride and hydrosulfide products.^254^ They also reported the activation of SF_6_ using a platinum complex, [Pt(PR_3_)_2_] (R = Cy, ^i^Pr), to generate *trans*-[Pt(F)(SF_3_)(PR_3_)_2_] (Scheme 8a). This complex was utilised in deoxyfluorination reactions of ketones, demonstrating the indirect use of SF_6_ as a fluorinating agent.^253^

Recent work has utilised organic derivatives and main group compounds for the selective degradation of SF_6_.^256,257^ Rueping and co-workers developed a method for the reduction of SF_6_ using bipyridine based organic reductants, to form ion pair products containing a donor dication and F^−^ and SF_5_^−^ anions. These salts were isolated and applied as fluorinating agents in the deoxyfluorination of alcohols, aldehydes and carboxylic acids.^257^ Dielmann and co-workers demonstrated the metal-free activation of SF_6_ by using a highly nucleophilic phosphine.^256^ By varying the phosphine ligands, SF_6_ was degraded to non-volatile phosphine sulfide and phosphine fluoride products, or in one case a bench-stable, crystalline SF_5_^−^ salt was isolated.^256^ These reactions were calculated to proceed *via* nucleophilic attack at the fluorine atom by the phosphine, in contrast to the previous SF_6_ degradation reports mentioned above involving alkali metals, organic reductants and transition-metal mediated transformations which were proposed to proceed by a single-electron transfer step to SF_6_.

A number of elegant photoredox strategies have been developed also.^258–262^ Notably, Braun and co-workers developed a system using an N-heterocyclic carbene (NHC) to reduce SF_6_ under photoredox conditions, forming a difluoroimidazolidine. This compound was then applied in the deoxyfluorination of a range of alcohols (Scheme 8b).^259^

**Scheme 9:**
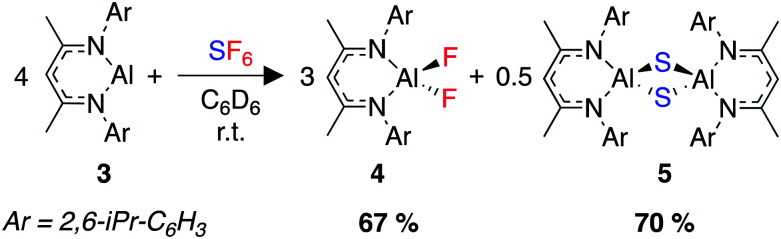
Room-temperature activation of SF_6_ using the aluminium(i) complex 3.

In 2021, our group sought to extend our previous work in the activation of environmentally persistent fluorocarbons to SF_6_. We developed a transition metal free reaction that rapidly reduces SF_6_ under mild conditions using a highly nucleophilic aluminium(i) complex, 3.^263^ The reaction produces two known, well-defined aluminium(iii) fluoride and sulfide complexes, 4 and 5 (Scheme 9). These two species are separable by virtue of their differing solubilities in the reaction solvent, with the sulfide species 5 forming a colourless precipitate from benzene or toluene.

**Fig. 4:**
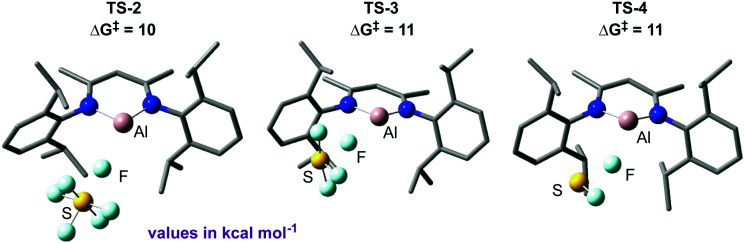
Key transition states calculated for the deconstruction of SF_6_ by 3.

Due to the limited mechanistic understanding of previous SF_6_ activation in the literature, we conducted an extensive computational study. The mechanism calculated suggests a series of nucleophilic steps involving the attack of 3 at the fluorine atom of an S–F bond, degrading SF_6_*via* SF_4_ and SF_2_ to the experimentally observed reaction products 4 and 5 (Fig. 4). Experimentally, no reaction intermediates were observed, and the reaction reaches completion within 15 minutes at room temperature, consistent with the small activation barriers calculated for each elementary step.

NBO analysis provided further evidence for a nucleophilic attack mechanism. Notably, an increasingly negative NPA charge at the sulfur atom was calculated as the transition state is traversed and conversely an increasingly positive NPA charge at aluminium. This suggests a transfer of electron density from aluminium to the sulfur atom. These calculations distinguished the proposed nucleophilic attack mechanism from a fluoride abstraction mechanism, where it would be expected that electron density would flow in the opposite direction. Extended Transition State-Natural Orbitals for Chemical Valence (ETS-NOCV) analysis was also carried out,^264^ and revealed the largest contribution to the orbital interaction between 1 and SF_6_ in TS-2 to be donation from the aluminium lone pair to σ*(S–F), with overlap occurring at the fluorine end of the bond.

**Scheme 10:**
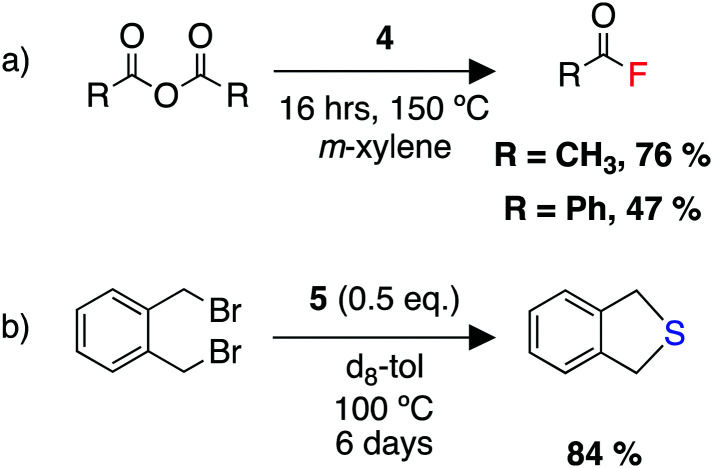
(a) Utility of 4 as a fluorinating agent for a range of electrophiles, (b) utility of 5 as a sulfinating agent.

We were able to demonstrate the utility of the products 4 and 5 as fluorinating and sulfinating agents (Scheme 10). Despite the high thermodynamic stability of the Al–F bond, a set of reactions were developed allowing transfer of the fluoride onto carbon, silicon, and boron electrophiles. The reaction with acid anhydrides allowed the formation of acyl fluorides. These compounds are of increasing importance as fluorinating agents in transition metal catalysed reactions, due to their unique balance of stability and reactivity.^265–267^ Overall, this process represents the repurposing of a waste greenhouse gas in the synthesis of compounds of value.

## 6. Conclusions and outlook

F-Gases have diverse uses across several important industries. Despite strong legislation that now controls many of these gases, data concerning emissions and atmospheric concentrations suggest that release of F-gases into the environment continues to be a problem. While several potential routes for the destruction of F-gases exist (thermal oxidation, catalytic hydrolysis, plasma treatment, electric discharge), most of these techniques are energy and cost intensive due to the extremely high temperatures required. They are often operationally difficult and carry the threat of producing hazardous decomposition products if conditions are not optimal. There is a further concern that some of these decomposition products may themselves contribute to environmental damage.

It is worthwhile considering that the C–F bonds within these waste fluorinated species are valuable, having been created by the fluorination of organic molecules likely using HF derived from inorganic sources. The repurposing of waste F-gases as a chemical feedstock to produce other useful fluorinated chemicals is a highly attractive alternative to destruction. The opportunity lies within chemistry to explore and develop methods for activating and functionalising the C–H and C–F bonds in these highly inert molecules. Although research is advancing, the development of chemical methods to upgrade inert substrates such as HFCs, PFCs and SF_6_ is difficult. In the presence of strong nucleophiles HFCs tend to react by pathways involving an initial deprotonation, while fluoride abstraction becomes more favourable in the presence of highly electrophilic Lewis acids. There is far less chemistry known for upgrading PFCs, but methods for the photochemical and thermal activation of SF_6_ are emerging.

The challenge in this field remains controlling the selectivity of these reactions to produce useful chemical building blocks for onward use. While several methods are emerging to use HFC-23 as a source of both –CF_3_ and –CF_2_H groups, HFC-134a and SF_6_ remain underexplored synthons for the creating –CH_2_CF_3_ and –SF_5_ groups. Significant technical obstacles remain for scaling-up and implementing these methods for real-life F-gas remediation. Flow methods are emerging as a powerful approach in this regard.^13^ The ultimate aim should be to employ chemical recycling methods using recovered F-gases. In the long-term it will be necessary to consider both life-cycle analysis and technoeconomic analysis of any technologies developed – as is already standard in CO_2_ remediation methods.

## Conflicts of interest

There are no conflicts to declare.

## Supplementary Material
